# A Robust Nonlinear Observer for Real-Time Attitude Estimation Using Low-Cost MEMS Inertial Sensors

**DOI:** 10.3390/s131115138

**Published:** 2013-11-06

**Authors:** José Fermi Guerrero-Castellanos, Heberto Madrigal-Sastre, Sylvain Durand, Lizeth Torres, German Ardul Muñoz-Hernández

**Affiliations:** 1 Faculty of Electronic, Autonomous University of Puebla (BUAP), Ciudad universitaria, Puebla 72570, Mexico; E-Mails: squall_mash@hotmail.com (H.M.-S.); gmunoz64@yahoo.co.uk (G.A.M.-H.); 2 Control Systems Department, GIPSA-lab laboratory, CNRS-University of Grenoble, ENSE3 BP 46, St Martin d'Hères Cedex 38402, France; E-Mail: sylvain@durandchamontin.fr; 3 Engineering Institute, Autonomous University of Mexico (UNAM), Circuito Escolar, Ciudad Universitaria, Mexico D.F. 04510, Mexico; E-Mail: ftorreso@iingen.unam.mx

**Keywords:** nonlinear attitude observer, quaternion, MEMS IMU, Attitude and Heading Reference System (AHRS), real-time implementation

## Abstract

This paper deals with the attitude estimation of a rigid body equipped with angular velocity sensors and reference vector sensors. A quaternion-based nonlinear observer is proposed in order to fuse all information sources and to obtain an accurate estimation of the attitude. It is shown that the observer error dynamics can be separated into two passive subsystems connected in “feedback”. Then, this property is used to show that the error dynamics is input-to-state stable when the measurement disturbance is seen as an input and the error as the state. These results allow one to affirm that the observer is “robustly stable”. The proposed observer is evaluated in real-time with the design and implementation of an Attitude and Heading Reference System (AHRS) based on low-cost MEMS (Micro-Electro-Mechanical Systems) Inertial Measure Unit (IMU) and magnetic sensors and a 16-bit microcontroller. The resulting estimates are compared with a high precision motion system to demonstrate its performance.

## 1. Introduction

### 1.1. Motivations and Background

The attitude control problem of a rigid body has attracted a strong interest during the past few decades, and it has been extensively studied. This interest comes from the fact that many aerospace systems, such as spacecrafts, satellites, tactical missiles and many others, enter within the framework of a rigid body with the requirement of precise attitude information [1]. In the last decade, the application of micro-electro-mechanical systems (MEMS) has gained a strong interest. Therefore, the attitude estimation problem has been tackled within new areas, such as terrestrial and aerial robotics [2,3], virtual reality [4], biomechanics [5] and wearable robots [6]. In these cases, the attitude information is obtained from inertial and magnetic sensors, namely, three rate gyros, three accelerometers and three magnetometers, orthogonally mounted, such that the sensor frame axes coincide with the principal axes of the rigid body. Since the attitude is not directly measured, it must be estimated via the measurements of the mentioned sensors. In general, these sensors can be classified into two main categories: *Angular velocity sensors* that measure the angular velocity of the body with respect to some inertial frame and *reference vector sensors* that give the coordinates of a fixed vector in the mobile frame.

Rate gyros are angular velocity sensors, and they provide continuous attitude information with good short-term stability when their measurements are integrated by means of the rigid body kinematic Equation. However, since rate gyro measurements are affected by drifts and noise, the attitude estimation based on these sensors diverges slowly from the real attitude. On the other hand, when reference vector sensors measurements, known as *vector observations*, are available, the attitude can be determinated by algebraic or optimization techniques [7]. Therefore, in order to decrease the effect of noise induced by the vector observations, as well as the attitude divergence provoked by the rate gyro drifts, it is important to fuse both information sources.

To this effect, various estimators have been proposed, using the quaternion attitude parametrization [8]. The Extended Kalman Filter (EKF) [9] and new alternatives to the standard EKF have been applied extensively (see [10] and the references therein). However, the divergence problem [11], the non-Gaussian noise induced [12] and the computational cost render difficult the embedded implementation of the Extended Kalman Filter (EKF). In recent years, significant research efforts have been addressed toward the synthesis of nonlinear attitude observers in order to tackle the previously mentioned issues. The first work on this topic was presented in [13]. Subsequently, in [14–16], quaternion-based nonlinear observers and gyro bias observers are proposed in the framework of satellite sensors calibration and marine vehicle navigation, respectively. Furthermore, the formulation of nonlinear attitude observers based on the the orthogonal group, *SO*(3), have been proposed in the few last years [17–20]. Recently, an excellent overview of rigid body attitude estimation and comparative study was given in [21,22]. Nevertheless, it is well known that a nonlinear observer is generally vulnerable to measurement disturbance, in the sense that a small arbitrary disturbance can lead to the divergence of the error state [23]. The above mentioned nonlinear attitude observers (with the exception of [20]) do not consider either the present noise in the gyro measurements nor a time-varying gyro bias term. Furthermore, the computational complexity in these approaches is a drawback, since the algorithms work with 3 × 3 matrices and they have to preserve orthogonality properties. The singular value decomposition (SVD) is a well-known approach and an extremely powerful and useful tool in linear control theory and signal processing. However, few works have exploited this approach in the framework of attitude estimation. In [24], the authors use the SVD method to estimate the attitude matrix minimizing Wahba's cost function. In the work reported in [25], the authors use the SVD approach for finding the relative orientation of a robotic manipulator. Nevertheless, the problem of attitude estimation from magnetic and inertial sensors using an SVD approach has not been addressed in the literature. Furthermore, unlike sensors and computer systems used in the marine and aerospace community, the signal output of the low-cost sensors is subject to high levels of noise and a time-varying bias term. These problems must be addressed, since, in a practical framework, the design of efficient and embedded attitude estimator using low-cost sensors is an important issue.

### 1.2. Contributions

In the present work, a quaternion-based attitude observer/estimator of a rigid body is presented. In the proposed approach, the attitude estimation problem is solved in two parts. Firstly, a quaternion attitude is estimated by means of vector observations. In this first step, the attitude estimation is performed using an SVD (singular value decomposition) approach. Then, the quaternion obtained in this step is considered an attitude measurement. Contrary to conventional techniques, the SVD maintains the quaternion's unit constraint naturally. Furthermore, the numerical robustness and numerical stability are guaranteed [26]. The second part of the proposed method consists of the design of a nonlinear observer in order to produce an estimate of the time-varying gyro bias and the attitude quaternion. This observer is driven by an attitude error obtained by means of the quaternion propagated by the observer, and this one obtained from the SVD technique. Asymptotic convergence of the estimation error is proven. Moreover, it is shown that the error dynamics can be decomposed in two passive subsystems connected in “feedback”. This result is exploited to prove that the observer is input-to-state stable (ISS) [27,28] when the rate gyro noise is seen as the input and the error as the state. In this sense, using the small gain theorem, one claims that the observer is “robustly stable”. To evaluate the proposed attitude observer behavior in real-time, a complete Attitude and Heading Reference System (AHRS) based on low-cost inertial and magnetic sensors and a 16-bit microcontroller is designed and implemented. A comparison with a high precision motion system is carried out, in order to demonstrate the observer performance.

The ISS paradigm in an attitude observer, the problem of estimating the attitude from vector observations using an SVD approach, as well as a real-time implementation have never been addressed in the literature. These facts show the originality of the present work.

The document is organized as follows. In Section 2, a mathematical background of the attitude parametrization and sensors modeling is given. The main problem is formulated in Section 3. The formulation of the nonlinear attitude observer and stability analysis is presented in Sections 4-6. The AHRS implementation and experimental results are given in Section 7. Finally, conclusions and further research are mentioned in Section 8.

## 2. Mathematical Background

### 2.1. Unit Quaternions and Attitude Kinematics

Consider two orthogonal, right-handed coordinate frames: the body coordinate frame, ${\text{E}}^{b}=[{\vec{e}}_{1}^{b},{\vec{e}}_{2}^{b},{\vec{e}}_{3}^{b}]$, located at the center of mass of the rigid body, and the inertial coordinate frame, ${\text{E}}^{f}=[{\vec{e}}_{1}^{f},{\vec{e}}_{2}^{f},{\vec{e}}_{3}^{f}]$, located at some point in the space. The rotation of the body frame, **E***^b^*, with respect to the fixed frame, **E***^f^*, is represented by the attitude matrix *R* ∈ *SO*(3) = { *R* ∈ ℝ^3×3^ : *R^T^R* = *I*, det *R* = 1}.

The cross product between two vectors, *χ⃗*, *ξ⃗* ∈ ℝ^3^, is represented by a matrix multiplication [*ξ⃗*^×^]*χ* = *ξ⃗* × *χ⃗*, where:

$$[{\vec{\xi }}^{\times }]=\left(\begin{array}{ccc} 0 & -\xi_{3} & \xi_{2}\\ \xi_{3} & 0 & -\xi_{1}\\ -\xi_{2} & \xi_{1} & 0 \end{array}\right)$$

The *n*-dimensional unit sphere embedded in ℝ *^n^*^+1^ is denoted as 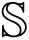
*^n^* = {*x* ∈ ℝ*^n+^*^1^: *x^T^x* = 1}. Members of *SO*(3) are often parametrized in terms of a rotation, *β* ∈ ℝ, about a fixed axis, *e⃗* ∈ 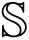
^2^, by the map, 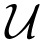 : ℝ × 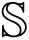
^2^ → *SO*(3), defined as:

(1) $$u(\beta ,\vec{e}):=I+\sin(\beta )[{\vec{e}}^{\times }]+(1-\cos(\beta )){\left[{\vec{e}}^{\times }\right]}^{2}$$

Hence, a unit quaternion, *q* ∈ 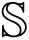
^3^, is defined as:

(2) $$q:=\left(\begin{array}{c} \cos\frac{\beta }{2}\\ \vec{e}\sin\frac{\beta }{2} \end{array}\right)=\left(\begin{array}{c} q_{0}\\ \vec{q} \end{array}\right)\in {\text{S}}^{3}$$

*q⃗* = [*q*_1_ *q*_2_ *q*_3_]*^T^* ∈ ℝ^3^ and *q*_0_ ∈ ℝ are known as the vector and scalar parts of the quaternion, respectively. *q* represents an element of *SO*(3) through the map, 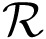 : 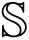
^3^ →*SO*(3), defined as:

(3) $$ℜ:=I+2q_{0}[{\vec{q}}^{\times }]+2{\left[{\vec{e}}^{\times }\right]}^{2}$$

Note that *R* = ( 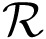) (*q*) = ( 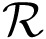) (—*q*) for each *q* ∈ 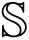
^3^, *i.e.*, quaternions *q* and –*q* represent the same physical attitude. The inverse unit quaternion is given by *q*^−1^ := [*q*_0_ –*q⃗^T^*]*^T^* , and the quaternion product is defined by:

(4) $$q_{1}⊗q_{2}:=\left(\begin{array}{cc} q_{1_{0}} & -{\vec{q}}_{1}^{T}\\ {\vec{q}}_{1} & I_{3}q_{1_{0}}+[{\vec{q}}_{1}^{\times }] \end{array}\right)\left(\begin{array}{c} q_{2_{0}}\\ {\vec{q}}_{2} \end{array}\right)$$

Denoting by *ω⃗* = [*ω*_1_ *ω*_2_ *ω*_3_]*^T^* the angular velocity vector of the the body coordinate frame, **E***^b^*, relative to the inertial coordinate frame, **E***^f^*, expressed in **E***^b^*, the kinematics equation is given by:

(5) $$\left(\begin{array}{c} {\dot{q}}_{o}\\ \dot{\vec{q}} \end{array}\right)=\frac{1}{2}\left(\begin{array}{c} -{\vec{q}}^{T}\\ I_{3}q_{0}+[{\vec{q}}^{\times }] \end{array}\right)\vec{\omega }=\frac{1}{2}\Xi (q)\vec{\omega }$$

The attitude error is used to quantify the mismatch between two attitudes. If *q* defines the true attitude quaternion and *q̂* is the estimated quaternion, then the error quaternion that represents the attitude error between the true orientation and the estimated one is given by [8]:

(6) $$q_{e}:={\hat{q}}^{-1}⊕q={\left(q_{e_{0}}{\vec{q}}_{e}^{T}\right)}^{T}$$

In the case that the true quaternion and the estimated one coincide, the quaternion error becomes:

(7) $$q_{e}={\left(\pm 1\;0^{T}\right)}^{T}$$

### 2.2. Sensor Modeling

The sensors in the rigid body are classified into the two following categories.

#### 2.2.1. Angular Velocity Sensors

The angular velocity *ω⃗* = (*ω*_1_ *ω*_2_ *ω*_3_)*^T^* is measured by three rate gyros orthogonally mounted. The output signal of a rate gyro is influenced by noise and by a slowly varying function, *v*, called bias [29], that is:

(8) $${\vec{\omega }}_{G}=\vec{\omega }+\vec{v}+{\vec{\eta }}_{G}$$

(9) $$\dot{\vec{v}}=-T^{-1}\vec{v}+{\vec{\eta }}_{v}$$

where *η⃗_G_* and *η⃗_v_* ∈ ℝ^3^ are bounded noises and *T* = *τI*_3_ is a diagonal matrix of time constants.

#### 2.2.2. Reference Vector Sensors

Let us consider the representation of a vector, *x⃗_i_*, with respect to **E***^f^* and **E***^b^* and denoted by *r⃗_i_* and *b⃗_i_*, respectively. The vectors, *r⃗_i_*, are also called the “reference vectors” and, in general, are known quite accurately. The body vectors, *b⃗_i_*, are known as “vector observations” and are obtained from on-board sensors (for instance, magnetometers, star trackers, accelerometers). Let *b_i_q__* and *r_i_q__* be the associate quaternion to the vectors, *b⃗_i_* and r⃗*_i_*:

(10) $$b_{i_{q}}={\left(0\;{\vec{b}}_{i}^{T}\right)}^{T}\;r_{i_{q}}={\left(0\;{\vec{r}}_{i}^{T}\right)}^{T}$$

these quaternions are related by the quaternion rotation, *q*, such that:

(11) $$b_{i_{q}}=q^{-1}⊗r_{i_{q}}⊗q$$

From Equation (4) and using quaternion algebra, the following measure model can be obtained [25]:

(12) $$\left(\begin{array}{cc} 0 & -{\left({\vec{b}}_{i}-{\vec{r}}_{i}\right)}^{T}\\ {\vec{b}}_{i}-{\vec{r}}_{i} & -[{\left({\vec{b}}_{i}+{\vec{r}}_{i}\right)}^{\times }] \end{array}\right)\;q=Hq=0$$

with *H* ∈ ℝ^4×4^. Note that Equation (12) is a well-structured linear system of equations.

## 3. Problem Statement

The objective is to design an attitude observer that estimates the rigid body attitude doing a trade-off between a good short-term precision given by rate gyro integration and a reliable long-term accuracy provided by vector observations.

If at least two vector observations are non-collinear, the quaternion can be estimated using these simultaneous vector measurements. For this purpose, the measurement Equation (12) is exploited. The observation matrix in a function of *m* pair vectors (*b⃗_i_*, *r⃗_i_*) with *i* ∈ {1, 2,…, *m*} is written as follows:

(13) $$\bar{H}=\left(\begin{array}{c} H_{1}\\ \vdots \\ H_{m} \end{array}\right)=\left(\begin{array}{cc} 0 & -{\left({\vec{b}}_{1}-{\vec{r}}_{1}\right)}^{T}\\ \left({\vec{b}}_{1}-{\vec{r}}_{1}\right) & -[{\left({\vec{b}}_{1}+{\vec{r}}_{1}\right)}^{\times }]\\ & \vdots \\ 0 & -{\left({\vec{b}}_{m}-{\vec{r}}_{m}\right)}^{T}\\ {\vec{b}}_{m}-{\vec{r}}_{m} & -[{\left({\vec{b}}_{m}+{\vec{r}}_{m}\right)}^{\times }] \end{array}\right)\in {\mathbb{R}}^{4m\times 4}$$

In the case of perfect measurements, the measure Equation *H̄_q_* = 0 is always satisfied. However, in practice, the measurements are polluted with perturbations originated from noise, vibrations, *etc*. Therefore, the problem consists of estimating the attitude quaternion from a perturbed observation matrix, *H̄*, that is:

(14) $$\begin{array}{c} q_{ps}=\arg\underset{q}{\min}\left\{\frac{1}{2}\;{\left\|{\bar{H}}_{q}\right\|}_{2}^{2}\right\}\\ \text{subject to}\;{\left\|q_{ps}\right\|}_{2}=1 \end{array}$$

On the other hand, if the initial value, *q*(*t*_0_), is known and the angular velocity measurements are perfect, *i.e.*, *ω⃗* = *ω⃗_G_*, it is possible to obtain *q*(*t*) by integrating the kinematics Equation (5). Nevertheless, *q*(*t*_0_) is, in general, unknown, furthermore, the angular velocities are polluted with bias and noise (see Equation (8)), which results in slow divergence of attitude over time. Therefore, the observation idea can be used to on-line correct the integration, *q̂*(*t*), of some erroneous *q̂* (*t*_0_) and attitude errors caused by the bias, and the noise presents in the rate gyro measurements. This correction can be carried out according to the measurable error *q_e_* = *q̂*^-1^ ⊗ *q_ps_*, where *q_ps_* is considered an attitude measure obtained by the vector observations.

The observation problem 1can then be formulated as follows: Given the kinematic equation of the rigid body and the model of time-varying bias evolution, find an estimate *q̂* (*t*) and *v̂* (*t*) from the knowledge of *ω_G_*(*t*) and *q_ps_*(*t*) for all *t* ≥ *t*_0_ ∈ ℝ_≥ 0_.

## 4. Nonlinear Observer Formulation

### 4.1. Attitude Estimation from Vector Observations

**Assumption 1.** *There are at least two linearly independent vectors, b⃗_i_ and b⃗_j_*, *with i* ≠ *j*, *that is, rank(H̄)* ≥ 3.

**Lemma 1**. *Let H̄* ∈ ℝ^4*m*×4^ *be the observation matrix formed by the m pair of vectors* (*b⃗_i_*, *r⃗_i_*) *with i* ∈ {1, 2, …,*m*}. *Then, there is an orthonormal matrix U* = (*U*_1_ *U*_2_ … *U*_4*m*_) ∈ ℝ^4*m*×4*m*^, *an orthonormal matrix V* = (*V*_1_ *V*_2_ *V*_3_ *V*_4_) ∈ ℝ^4×4^ *and an orthonormal matrix S* ∈ ℝ^4*m*×4^, *with elements σ_k_ along the diagonal and zeros everywhere else, where k* = 1 : 4, *such that:*

(15) $$\bar{H}=USV^{T}$$

*Then, the attitude quaternion is given by the last column of the matrix, V, that is:*

(16) $$q_{ps}=V_{4}$$

*furthermore, the normality condition* ‖*q_ps_*‖_2_ = 1 *is satisfied*.

*Proof.* The proof follows the standard least squares solution of homogeneous equations via the SDV approach [30].

### 4.2. Nonlinear Attitude Observer with Bias Estimation

The proposed attitude nonlinear observer that includes the bias and the error update is given by:

(17) $$O_{A}:\{\begin{array}{l} \dot{\hat{q}}=\frac{1}{2}\Xi (\hat{q})({\vec{\omega }}_{G}-\hat{\vec{v}}+K_{1}{\vec{q}}_{e})\\ \dot{\hat{\vec{v}}}=-T^{-1}\hat{\vec{v}}-K_{2}{\vec{q}}_{e} \end{array}$$

where *T* ∈ ℝ^3×3^ has been defined in Equation (9) and *K*_1_, *K*_2_ are positive constant parameters. *q̂* is the prediction of the attitude at time *t*. It is obtained via the integration of the kinematics Equation (17), using the measured angular velocity, *ω⃗_G_*, the bias estimate, *ν̂* and *q⃗_e_*, which is the vector part of the quaternion error, *q_e_*, that measures the discrepancy between *q̂* and the measured attitude, *q_ps_*, obtained by means of Lemma 1, that is:

(18) $$q_{e}={\hat{q}}^{-1}⊗q_{ps}={\left(q_{e_{o}}\;{\vec{q}}_{e}^{T}\right)}^{T}$$

**Figure 1.**
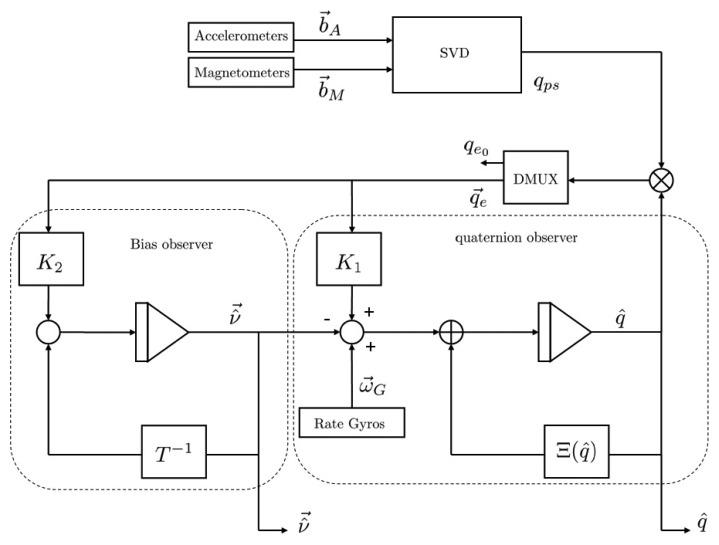
Block diagram of the nonlinear observer (⊗ quaternion multiplication, ⊗ matrix multiplication.)

Actually, the observer Equation (17) allows one to fuse the data that arise from the vector observations and the rate gyro measurements. The observer structure is shown in Figure 1.

Note that the attitude quaternion, *q_ps_*, can be expressed by:

(19) $$q_{ps}=q⊗\Delta q$$

where *q* denotes the true attitude and Δ*q* represents a perturbation quaternion, which is caused by the sensor measurement noise encountered in practice.

For the first time, a Lyapunov stability analysis is carried out. As is always done, the error dynamic is obtained in a noise-free context; then, the following hypotheses are made:

● *η⃗_G_* = *η⃗_v_*= 0. The measurement rate gyro noise is not included in the error dynamics.
● Δ*q* = (1 0 0 0)^T^, *i.e.*, *q* = *q_ps_*. This is a good assumption for the case of static attitudes and for quasistatic movements [17].

Combining Equations (5)-(9) and (17), the error dynamics is expressed as:

(20) $$\sum_{e}:\{\begin{array}{l} {\dot{q}}_{e}=\frac{1}{2}\;\left(\begin{array}{cc} 0 & {\vec{\gamma }}^{T}\\ -\vec{\gamma } & \left[2{\vec{\omega }}^{\times }]+[{\vec{\gamma }}^{\times }\right] \end{array}\right)\;\left(\begin{array}{c} q_{e_{0}}\\ {\vec{q}}_{e} \end{array}\right)\\ \dot{\tilde{v}}=-T^{-1}\tilde{v}+K_{2}\;{\vec{q}}_{e} \end{array}$$

where *γ⃗* = *ṽ* + *K*_1_*q⃗_e_* and $\tilde{v}=\vec{v}-\hat{\vec{v}}$.

The system Equation (20) admits a set of equilibrium points 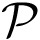 := {*p_e_*_+_, *p_e_*_−_}, where:

● *p_e_*_+_ :=(1 0*^T^* 0*^T^*)*^T^* ∈ 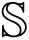
^3^ × ℝ^3^
● *p_e_*_−_ := (-1 0*^T^* 0*^T^*)*^T^* ∈ 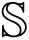
^3^ × ℝ^3^

In both scenarios, one shows that the following stability property is ensured with respect to different sets.

**Definition 1.** *(Asymptotic Stability in the Large)* [31]. *Let χ* ⊂ ℝ*^n^ be given. The trivial solution x* = 0 *of ẋ* = *f*(*t*, *x*) *is called asymptotically stable in the large with respect to χ if it is stable in the sense of Lyapunov and every other solution x*(*t*, *t*_0_, *x*_0_) → 0 *as t* → ∞ *for any initial states, x*_0_∈ *χ*, *and for any initial times, t*_0_∈ ℝ _≥0_.

One considers that the state space of Equation (20) corresponds to either *χ_+_* = {*q_e_* ∈ 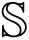
^3^|*q_e_0__* ∈ [0, 1]} × ℝ^3^ or *χ_* = {*q_e_* ∈ 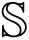
^3^|*q_e_0__* ∈ [-1, 0]} × ℝ^3^, depending on which target equilibrium is chosen.

**Proposition 1.** *Assume that all trajectories with initial conditions*, ( ${q_{e_{o}}(t_{0}){\vec{q}}_{e}^{T}(t_{0}){\tilde{v}}^{T}(t_{0}))}^{T}\in \chi_{+}$, *satisfy sgn*(*q_e_0__*(*t*)) = *sgn*(*q_e_0__*(*t*_0_)) ≥ 0 *for all t* > *t*_0_ (*mutatis mutandis for χ*_–_). *Then, the equilibrium point, p_e_*_+_ (*respectively, the equilibrium point, p_e_*_−_) *of the system Equation (20) is asymptotically stable in the large with respect to χ_+_* (*respectively, with respect to χ_–_*).

*Proof.* Consider the candidate Lyapunov function, *V* : 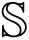
^3^ × ℝ*^3^* → ℝ, which is proper and positive definite:

(21) $$V=K_{2}({\left(1-q_{e_{0}}\right)}^{2}+{\vec{q}}_{e}^{T}{\vec{q}}_{e})+\frac{1}{2}{\tilde{v}}^{T}\tilde{v}=2K_{2}(1-q_{e_{0}})+\frac{1}{2}{\tilde{v}}^{T}\tilde{v}$$

The derivative of Equation (21), together with Equation (20), is given by:

(22) $$\begin{array}{ll} \dot{V} & =-2K_{2}{\dot{q}}_{e_{0}}+{\tilde{v}}^{T}\dot{\tilde{v}}=-K_{2}{\vec{\gamma }}^{T}{\vec{q}}_{e}+{\tilde{v}}^{T}(-T^{-1}\tilde{v}+K_{2}{\vec{q}}_{e})\\ & =-K_{2}({\tilde{v}}^{T}+K_{1}{\vec{q}}_{e}^{T}){\vec{q}}_{e}-{\tilde{v}}^{T}T^{-1}\tilde{v}+K_{2}{\tilde{v}}^{T}{\vec{q}}_{e}=-K_{2}K_{1}{\vec{q}}_{e}^{T}{\vec{q}}_{e}-{\tilde{v}}^{T}T^{-1}\tilde{v}\leq 0 \end{array}$$

Thus, *q⃗_e_→* 0 and *ṽ* → 0 and, due to the normality condition, *q_e_0__* → 1. Hence, solutions of the system Equation (20) whose initial conditions are in *χ*_+_ converge asymptotically to *p_e_*_+_ := (1 0*^T^* 0*^T^*)*^T^* . That concludes the proof.

**Remark 1.** *In a practical context, one aims at improving the convergence speed of the observer. Since, physically, the set of equilibrium points*, 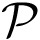 := {*p_e_*_+_,*p_e_*_−_}, *corresponds to the same attitude error, it is necessary to establish the desired equilibrium point to be achieved, depending on the given initial condition. This improvement can be ensured by choosing the equilibrium point corresponding to the sign of q_e_0__*(*t*_0_). *The point, p_e_*_+_, *is chosen if q_e_0__*(*t*_0_) ≥ 0 *and the point, p_e_*_−_, *otherwise. Then, a slight modification on the observer Equation (17) is done, obtaining*

(23) $$O_{A_{dis}}:\{\begin{array}{l} \dot{\hat{q}}=\frac{1}{2}\Xi (\hat{q})({\vec{\omega }}_{G}-\hat{\vec{v}}+\mathrm{sign}(q_{e_{o}})K_{1}{\vec{q}}_{e})\\ \dot{\hat{\vec{v}}}=-T^{-1}\hat{\vec{v}}-\mathrm{sign}(q_{e_{o}})K_{2}\;{\vec{q}}_{e} \end{array}$$

*This fact can be shown adapting the proof of Proposition 1 using the following Lyapunov function instead:*

(24) $$V=\{\begin{array}{c} K_{2}({\left(1-q_{e_{0}}\right)}^{2}+{\vec{q}}_{e}^{T}{\vec{q}}_{e})+\frac{1}{2}{\tilde{v}}^{T}\tilde{v},if\;q_{e_{0}}(t_{0})\geq 0\\ K_{2}({\left(1+q_{e_{0}}\right)}^{2}+{\vec{q}}_{e}^{T}{\vec{q}}_{e})+\frac{1}{2}{\tilde{v}}^{T}\tilde{v},if\;q_{e_{0}}(t_{0})<0 \end{array}$$

### 5. Passivity Interpretation of the Attitude Nonlinear Observer

**Lemma 2.** *(Kalman-Yakubovich-Popov (KYP) lemma) Let Z*(*s*) = *C*(*sI – A*)^−1^*B be a p* × *p transfer function matrix, where A is Hurwitz*, (*A*, *B*) *is controllable and* (*A*, *C*) *is observable. Then, Z*(*s*) *is strictly positive real (SPR) if and only if there exist a positive definite symmetric matrix P* = *P^T^* > 0 *and Q* = *Q^T^* > 0, *such that:*

(25) $$A^{T}P+PA=-Q$$

(26) $$B^{T}P=C$$

Since *K*_2_ is a scalar, the dynamics error Equation (20) for the bias observer can be written as:

(27) $$\dot{\tilde{v}}=-T^{-1}\tilde{v}+K_{2}I_{3}{\vec{q}}_{e}$$

Although the error system Equation (27) has no output, it is possible to define the following virtual output:

(28) $${\vec{y}}_{e}=K_{2}I_{3}\tilde{v}$$

**Figure 2.**
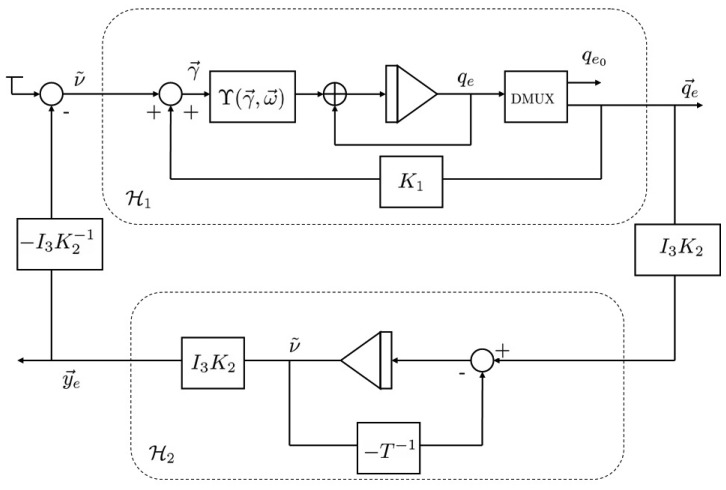
Block diagram of the dynamics of attitude and bias error.

Using Equation (28), the error dynamics Equation (20) can be structured as the one shown in Figure 2.

**Proposition 2.** *The transfer function matrix of the error system Equations (27) and (28) is SPR. That is, the mapping, q⃗_e_* → *y⃗_e_*, *satisfies the KYP lemma*.

*Proof.* Since the matrix, –*T*^−1^, is symmetric and a Hurwitz, one has for all *Q* symmetric positive definite matrices a *P* symmetric positive definite matrix, such that:

(29) $${\left(-T^{-1}\right)}^{T}P+P(-T^{-1})=-Q$$

If the *Q* matrix is specified as *Q* = 2*T*^−1^, one gets:

(30) $$P=I_{3}$$

then,

(31) $$B^{T}P={\left(K_{2}I_{3}\right)}^{T}I_{3}=K_{2}I_{3}=C$$

which satisfies the equality Equation (26).

**Proposition 3.** *The dynamics of the quaternion error, represented by the system*, 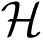
_1_, *is state strictly passive*.

*Proof.* Consider the candidate Lyapunov function, *V_q_*, which is positive, definite and proper:

(32) $$V_{q}=\{\begin{array}{l} K_{2}({\left(1-q_{e_{0}}\right)}^{2}+{\vec{q}}_{e}^{T}{\vec{q}}_{e})=2K_{2}(1-q_{e_{0}}),si\;q_{e_{0}}\geq 0\\ K_{2}({\left(1+q_{e_{0}}\right)}^{2}+{\vec{q}}_{e}^{T}{\vec{q}}_{e})=2K_{2}(1+q_{e_{0}}),si\;q_{e_{0}}<0 \end{array}$$

Analyzing for *q_e_0__* ≥ 0, the derivative of Equation (32) along the trajectories of *q_e_* (Equation (20)), is given by:

(33) $$\begin{array}{ll} {\dot{V}}_{q} & =-2K_{2}{\dot{q}}_{e_{0}}=-K_{2}{\vec{\gamma }}^{T}{\vec{q}}_{e}\\ & =-K_{2}({\tilde{v}}^{T}+K_{1}{\vec{q}}_{e}^{T}){\vec{q}}_{e}=-K_{2}K_{1}{\vec{q}}_{e}^{T}{\vec{q}}_{e}-K_{2}{\tilde{v}}^{T}{\vec{q}}_{e} \end{array}$$

or, similarly:

(34) $$-K_{2}{\tilde{v}}^{T}{\vec{q}}_{e}={\dot{V}}_{q}+K_{2}K_{1}{\vec{q}}_{e}^{T}{\vec{q}}_{e}$$

Since *q_e_0__* ∈ [0, 1], one obtains:

(35) $${\vec{q}}_{e}^{T}{\vec{q}}_{e}=1-q_{e_{0}}^{2}\geq 1-q_{e_{0}}\geq {\left(1-q_{e_{0}}\right)}^{2}$$

Then:

(36) $$\underset{\text{Power Flow}}{\underset{︸}{K_{2}{\tilde{v}}^{T}{\vec{q}}_{e}}}\geq \underset{\text{Rate of change of energy stored}}{\underset{︸}{{\dot{V}}_{q}}}+\underset{\text{State dissipation rate}}{\underset{︸}{K_{2}K_{1}{\left(1-q_{e_{0}}\right)}^{2}}}$$

**Theorem 1.** *The attitude nonlinear observer Equation (17) is passive*.

*Proof.* Propositions 2 and 3 establish that the systems, 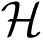
_2_ and 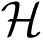
_1_, are SPR and state strictly passive, respectively. Since the two systems are connected in feedback (see Figure 2), the attitude nonlinear observer Equation (17) is passive.

## 6. Input to State Stability (ISS) of the Attitude Nonlinear Observer

Note that the convergence and the passivity interpretation of the observer were accomplished within a context free from noise and perturbation. However, the rate gyro measurements are corrupted by measurements noise and bias components that are time-varying. Owing to the nonlinearity of the observer, this type of disturbance can cause instability or even the finite time escape of the estimate.

In this section, the robustness of the proposed observer is discussed, which follows from passivity propriety. It will be shown that the error dynamics is input-to-state stable when the disturbances are viewed as the input and the error quaternion as the state.

In the case that noise is included, the error dynamics Equation (20) becomes:

(37) $$\sum_{e_{\text{noise}}}:\{\begin{array}{l} {\dot{q}}_{e}=\frac{1}{2}\left(\begin{array}{cc} 0 & {\vec{\gamma }}^{T}+{\vec{\eta }}_{G}^{T}\\ -\vec{\gamma }+{\vec{\eta }}_{G} & \left[2{\vec{\omega }}^{\times }]+[{\left(\vec{\gamma }+{\vec{\eta }}_{G}\right)}^{\times }\right] \end{array}\right)\;\left(\begin{array}{c} q_{e_{0}}\\ {\vec{q}}_{e} \end{array}\right)\\ \dot{\tilde{v}}=-T^{-1}\tilde{v}+K_{2}\;{\tilde{q}}_{e}+{\vec{\eta }}_{v} \end{array}$$

**Figure 3.**
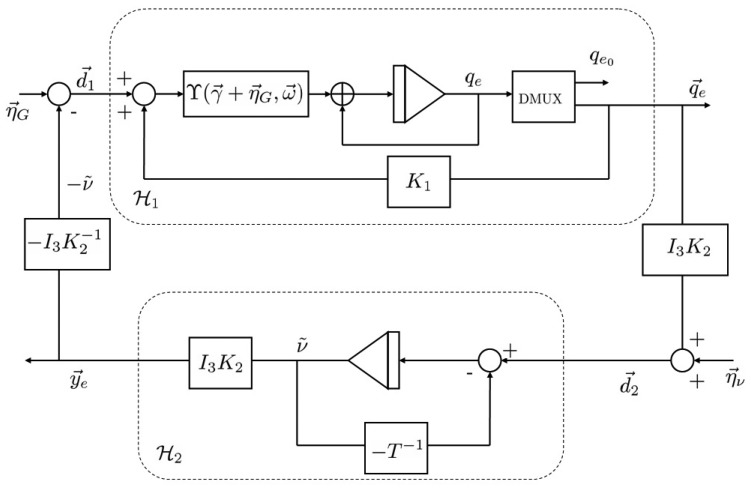
Block diagram of the dynamics of attitude and bias error with measurement disturbances.

This equation can be described as illustrated in Figure 3, where the inputs, *d⃗*_1_ and *d⃗_2_*, are such that:

$${\vec{d}}_{1}=\tilde{v}+{\vec{\eta }}_{G},\;{\vec{d}}_{2}=K_{2}{\vec{q}}_{e}+{\vec{\eta }}_{v}$$

It is assumed that the noise components are bounded. That is:

(38) $$\begin{array}{cc} \underset{s\in [t_{0},t]}{\sup}{\left\|{\vec{\eta }}_{G}(s)\right\|}_{2}<c_{1} & \underset{s\in [t_{0},t]}{\sup}{\left\|{\vec{\eta }}_{v}(s)\right\|}_{2}<c_{2} \end{array}$$

However, neither the bounds nor the noise distribution are necessarily known.

**Proposition 4.** *The system*, 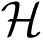
_2_, *is input-to-state stable (ISS) with respect to the input, d⃗*_2_. *Then, the output of* 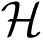
_2_ (*y⃗_e_* = *K*_2_*I*_3_*ṽ) is always bounded*.

*Proof.* Consider the second equation of the system Equation (37). Since the matrix, –*T*^−1^, is a Hurwitz, one obtains:

(39) $$\begin{array}{ll} {\left\|\tilde{v}(t)\right\|}_{2} & =e^{-T^{-1}}(t-t_{0}){\left\|\tilde{v}(t_{0})\right\|}_{2}+\int_{t_{0}}^{t}e^{-T^{-1}(t-s))}{\vec{d}}_{2}(s)ds\\ & =e^{-T^{-1}(t-t_{0})}{\left\|\tilde{v}(t_{0})\right\|}_{2}+\int_{t_{0}}^{t}e^{-T^{-1}(t-s)}(K_{2}{\vec{q}}_{e}(s)+{\vec{\eta }}_{v}(s))ds\\ & \leq e^{-T^{-1}(t-t_{0})}{\left\|\tilde{v}(t_{0})\right\|}_{2}+K_{2}\underset{s\in [t_{0},t]}{\sup}\{{\left\|{\vec{q}}_{e}(s)\right\|}_{2}\}+\underset{s\in [t_{0},t]}{\sup}\{{\left\|{\vec{\eta }}_{v}(s)\right\|}_{2}\} \end{array}$$

then:

(40) $${\left\|\tilde{v}(t)\right\|}_{2}\leq \max\{\zeta_{2}({\left\|\tilde{v}(t_{0})\right\|}_{2},t-t_{0}),\underset{s\in [t_{0},t]}{\sup}\varsigma_{21}({\left\|{\vec{q}}_{e}(s)\right\|}_{2}),\underset{s\in [t_{0},t]}{\sup}\varsigma_{2}({\left\|{\vec{\eta }}_{v}(s)\right\|}_{2})\}$$

where: *ζ*(‖*ṽ*(*t*_0_)‖_2_,(*t* − *t*_0_) = *e*^−^*^T^*^−1(^*^t^*^−^*^t^*^_0_)^‖*ṽ*(*t*_0_)‖_2_ is a 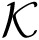
ℒ function and *ς*_21_ (‖*q⃗_e_*(*s*)‖_2_) = *K*_2_‖*q⃗_e_*(*s*)‖_2_ and *ς*_2_(‖*η⃗_v_*(*s*)‖_2_) = ‖*η⃗_v_*(*s*)‖_2_ are 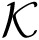 functions. Then, the bias estimation error, *ṽ*(*t*), is input-to-state stable, and the output *y⃗_e_*(*y⃗_e_* = *K*_2_*I*_3_*ṽ*) is always bounded.

**Proposition 5.** *The system*, 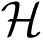
_1_, *is input-to-state stable with respect to d⃗*_2_.

The key to showing the ISS of the system, 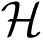
_1_, is the fact that a system is input-to-state stable if it admits an ISS-Lyapunov function. In this sense, a Lyapunov function will be proposed; then, it will be shown that this function is a ISS-Lyapunov function.

*Proof.* Consider the candidate Lyapunov function, *V*, which is proper and positive definite:

(41) $$V_{q}=\{\begin{array}{c} K_{2}\left({\left(1-q_{e_{0}}\right)}^{2}+{\vec{q}}_{e}^{T}{\vec{q}}_{e}\right)=2K_{2}\left(1-q_{e_{0}}\right),si\;q_{e_{0}}\geq 0\\ K_{2}\left({\left(1+q_{e_{0}}\right)}^{2}+{\vec{q}}_{e}^{T}{\vec{q}}_{e}\right)=2K_{2}\left(1+q_{e_{0}}\right),si\;q_{e_{0}}<0 \end{array}$$

Analyzing for *q_e_0__* ≥ 0, the derivative of Equation (32) along the trajectories of *q_e_* (Equation (37)) is given by:

(42) $$\begin{array}{ll} {\dot{V}}_{q} & =-2K_{2}{\dot{q}}_{e_{0}}\\ & =-K_{2}\left({\vec{\gamma }}^{T}+{\vec{\eta }}_{G}^{T}\right){\vec{q}}_{e}\\ & =-K_{2}\left({\tilde{\nu }}^{T}+K_{1}{\vec{q}}_{e}^{T}+{\vec{\eta }}_{G}^{T}\right){\vec{q}}_{e}\\ & =-K_{2}K_{1}{\vec{q}}_{e}^{T}{\vec{q}}_{e}-K_{2}\left({\tilde{\nu }}^{T}+{\vec{\eta }}_{G}^{T}\right){\vec{q}}_{e}\\ & =-K_{2}K_{1}{\vec{q}}_{e}^{T}{\vec{q}}_{e}-K_{2}{\vec{d}}_{1}^{T}{\vec{q}}_{e} \end{array}$$

since *q_e_0__* ∈ [0,1], one obtains:

(43) $${\vec{q}}_{e}^{T}{\vec{q}}_{e}={\left\|{\vec{q}}_{e}\right\|}_{2}^{2}=1-q_{e_{0}}^{2}\geq 1-q_{e_{0}}\geq {\left(1-q_{e_{0}}\right)}^{2}$$

then:

(44) $$\begin{array}{ll} {\dot{V}}_{q} & \leq -K_{2}K_{1}{\left(1-q_{e_{0}}\right)}^{2}-{\vec{d}}_{1}^{T}{\vec{q}}_{e}\\ & \leq -K_{2}K_{1}{\left(1-q_{e_{0}}\right)}^{2}+{\left\|{\vec{d}}_{1}\right\|}_{2}{\left\|{\vec{q}}_{e}\right\|}_{2}\\ & \leq -K_{2}K_{1}{\left(1-q_{e_{0}}\right)}^{2}+{\left\|{\vec{d}}_{1}\right\|}_{2}\\ & \leq -\alpha_{3}\left({\left(1-q_{e_{0}}\right)}^{2}\right)+\alpha_{4}\left({\left\|{\vec{d}}_{1}\right\|}_{2}\right) \end{array}$$

since *V_q_* is proper ∃ *α*_1_, *α*_2_ 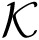
_∞_ functions, such that:

$$\alpha_{1}\left({\left\|q\left(t_{0}\right)\right\|}_{2}\right)\leq V_{q}\left(q\left(t_{0}\right)\right)\leq \alpha_{2}\left({\left\|q\left(t_{0}\right)\right\|}_{2}\right)$$

Furthermore, *α*_3_ ((1 – *q_e_0__*)^2^) = *K*_1_*K*_2_((1 – *q_e_0__*)^2^ and *α*_3_((1−*q_e_0__*)^2^ = *K*_1_*K*_2_)((1−*q_e_0__*)) are 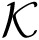
_∞_ functions. Then, the Lyapunov function, *V_q_*, is a ISS-Lyapunov function (see [27]), and this fact implies that the system, 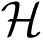
_1_, is ISS.

Since the system, 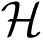
_1_, is ISS, the following inequality is accomplished:

(45) $$\begin{array}{ll} {\left\|{\vec{q}}_{e}\left(t\right)\right\|}_{2} & \leq \max\left\{\zeta_{1}\left({\left\|{\vec{q}}_{e}\left(t_{0}\right)\right\|}_{2},t-t_{0}\right),\underset{s\in \left[t_{0},t\right]}{\sup}\varsigma_{d}\left({\left\|{\vec{d}}_{1}\left(s\right)\right\|}_{2}\right)\right\}\\ & \leq \max\left\{\zeta_{1}\left({\left\|{\vec{q}}_{e}\left(t_{0}\right)\right\|}_{2},t-t_{0}\right),\underset{s\in \left[t_{0},t\right]}{\sup}\varsigma_{12}\left({\left\|\tilde{\nu }\left(s\right)\right\|}_{2}\right),\underset{s\in \left[t_{0},t\right]}{\sup}\varsigma_{1}\left({\left\|{\vec{\eta }}_{G}\left(s\right)\right\|}_{2}\right)\right\} \end{array}$$

where *ζ*_1_ is a 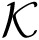
ℒ function and *ζ*_12_(‖*ṽ*‖_2_) = *K*_2_‖*ṽ*‖_2_ and *ζ_1_*(‖*η⃗_G_* ‖_2_) = *K*_2_‖ *η⃗_G_* ‖_2_ are 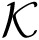 functions. That is, every trajectory of the attitude error dynamics asymptotically approaches a ball around the origin, whose radius is a function of supremum norm of the bias estimation error and of the measure noise of the rate gyro.

Using the interesting properties of ISS systems, the stability of the entire error dynamics is studied. Then, one has the following result.

**Theorem 2.** *Let K*_2_ *be a positive constant, such that K*_2_ < 1. *Then, the attitude nonlinear observer is robust towards to measurement disturbance of the rate gyro*.

*Proof.* The claim follows from the small gain theorem for the interconnection of nonlinear systems that are input-to-state stable [32]. The error dynamics of a nonlinear observer (Equation (37)) is composed of the feedback interconnection of the systems 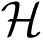
_1_- 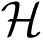
_2_). From Propositions 4 and 5, it follows that:

(46) $${\left\|{\vec{q}}_{e}\left(t\right)\right\|}_{2}\leq \max\left\{\zeta_{1}\left({\left\|{\vec{q}}_{e}\left(t_{0}\right)\right\|}_{2},t-t_{0}\right),\underset{s\in \left[t_{0},t\right]}{\sup}\varsigma_{12}\left({\left\|\tilde{\nu }\left(s\right)\right\|}_{2}\right),\underset{s\in \left[t_{0},t\right]}{\sup}\varsigma_{1}\left({\left\|{\vec{\eta }}_{G}\left(s\right)\right\|}_{2}\right)\right\}$$

(47) $${\left\|\tilde{\nu }\left(t\right)\right\|}_{2}\leq \max\left\{\zeta_{2}\left({\left\|\tilde{\nu }\left(t_{0}\right)\right\|}_{2},t-t_{0}\right),\underset{s\in \left[t_{0},t\right]}{\sup}\varsigma_{21}\left({\left\|{\vec{q}}_{e}\left(s\right)\right\|}_{2}\right),\underset{s\in \left[t_{0},t\right]}{\sup}\varsigma_{2}\left({\left\|{\vec{\eta }}_{\nu }\left(s\right)\right\|}_{2}\right)\right\}$$

The composition of the functions, *ς*_12_ and *ς*_21_, is given by:

(48) $$\varsigma_{12}\circ \varsigma_{21}\left({\left\|{\vec{q}}_{e}\left(s\right)\right\|}_{2}\right)=K_{2}\left(K_{2}{\left\|{\vec{q}}_{e}\right\|}_{2}\right)$$

Since *K_2_* < 1, it follows that:

(49) $$K_{2}^{2}{\left\|{\vec{q}}_{e}\right\|}_{2}<{\left\|{\vec{q}}_{e}\right\|}_{2}\forall {\left\|{\vec{q}}_{e}\right\|}_{2}>0$$

Then, the system ( 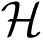
_1_- 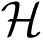
_2_) is ISS, when the measurements disturbances, *η⃗_G_* and *η⃗_v_*, are seen as the input and the error state, [*q⃗_e_^T^ṽ^T^*]*^T^*, as the state. This means that the attitude observer is robust with respect to measurement disturbance.

## 7. Experimental Results

The estimation methodology proposed in this work is implemented and evaluated in real time, in order to assess its effectiveness. For this purpose, an embedded system was designed and developed. Special attention was paid to the low power consumption requirements and weight, leading to the selection of the digital signal controller (DSC), dsPIC33FJ128MC802, which was used with a clock speed of 4 MHz. It contains extensive digital signal processor (DSP) functionality with a high performance, 16-bit microcontroller (MCU) architecture, but without a floating point unit. The sensor suite is based on a sensor board equipped with a tri-axis accelerometer (ADXL135), a dual axis gyro (LPR530AL), a single axis gyro (LY530ALH) and a tri-axis magnetometer (Micromag 3). All sensors outputs are analog, except for the Micromag 3, which is digital and uses the serial peripheral interface (SPI) bus system as the underlying physical communication layer. The aim was to test the DSC implementation with an update rate of the estimation of at least 55 Hz.

**Figure 4.**
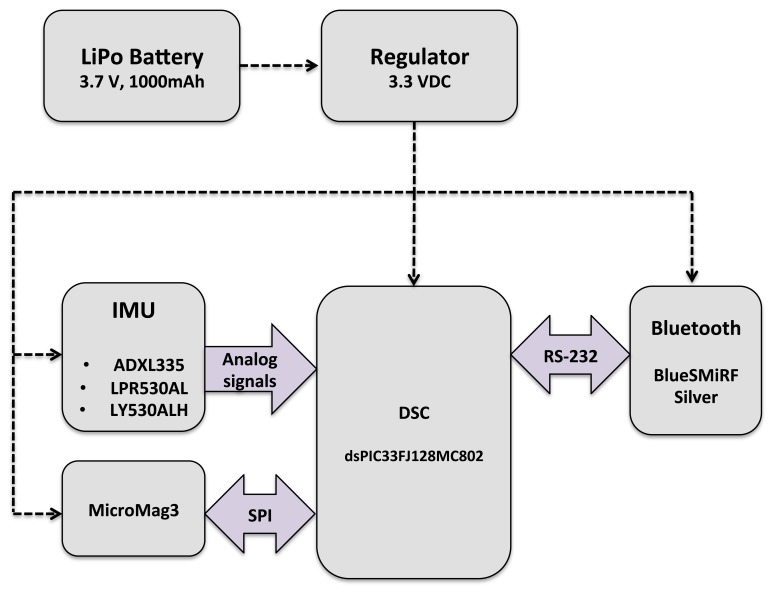
Block diagram for the Attitude and Heading Reference System (AHRS) prototype.

**Figure 5.**
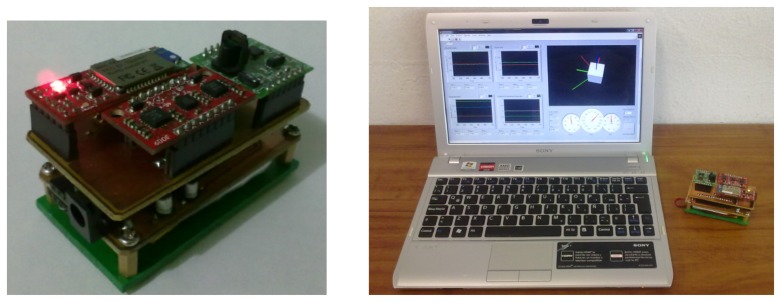
The AHRS prototype.

Furthermore, the system is equipped with a Bluetooth module (BlueSMiRF Silver), which provides wireless capabilities. The total system supply voltage is 3.3 V. The dimension and weight are 60 × 40 × 15 mm and 60 g, respectively. The system is depicted in Figures 4 and 5.

**Figure 6.**
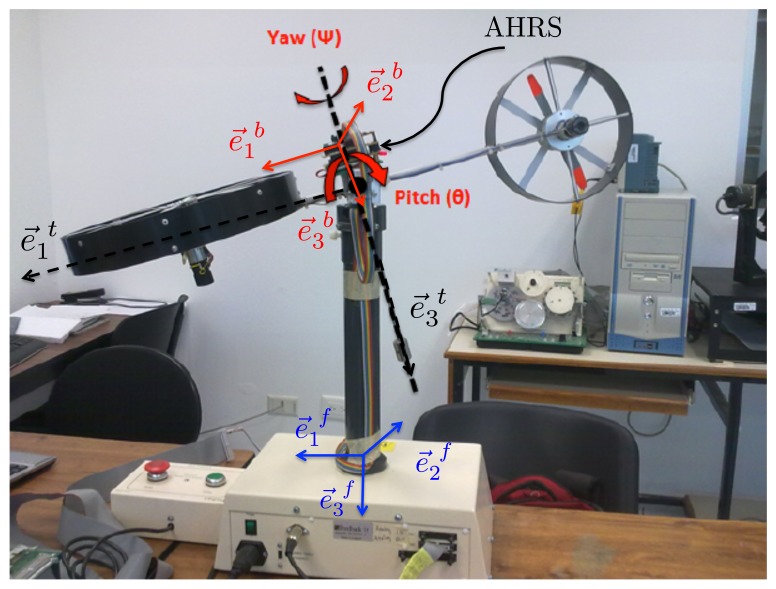
Experimental setup.

In order to experimentally evaluate the performance of the proposed AHRS, the experiments were carried out with a two degrees-of-freedom high accuracy system. Actually, this system is the Twin Rotor System [33] (see Figure 6). This system has two rotational joints, which allow for movement about two orthogonally axes with a resolution of 0.35° considered a true attitude for performance evaluation. In order to test the complete attitude estimation, two trials were carried out. In the first one, the axes, ${\vec{e}}_{1}^{b}$ and ${\vec{e}}_{3}^{b}$, of the AHRS coordinate frame (body coordinate frame ${\text{E}}^{b}=[{\vec{e}}_{1}^{b},{\vec{e}}_{2}^{b},{\vec{e}}_{3}^{b}]$) coincide with the axes, ${\vec{e}}_{1}^{t}$ and ${\vec{e}}_{3}^{t}$, of the Twin Rotor System. Thus, *θ* (pitch) and *Ψ* (yaw) angles were evaluated. In order to evaluate the angle, *Φ* (roll), the AHRS was rotated *π*/2 about the axis, ${\vec{e}}_{1}^{b}$, such that ${\vec{e}}_{1}^{b}$ coincides with ${\vec{e}}_{1}^{t}$ and ${\vec{e}}_{2}^{b}$ with ${\vec{e}}_{3}^{t}$. Note that the attitude is estimated with respect to the inertial coordinate frame ${\text{E}}^{f}=[{\vec{e}}_{1}^{f},{\vec{e}}_{2}^{f},{\vec{e}}_{3}^{f}]$. **E***^f^* is chosen to be the NED (north, east, down) coordinate frame. In this case, the reference vectors are the gravitational and magnetic vectors. The vector observations, *i.e.*, the gravitational and magnetic vectors expressed in the body frame, **E***^b^*, are obtained from the mentioned tri-axis accelerometer and tri-axis magnetometer. The angular velocity is obtained from three rate gyros.

The attitude and gyro bias observer is implemented at 55 Hz using a fourth-order Runge-Kutta method, with tuning parameters, *K*_1_ = 3.2 and *K*_2_ = 0.9, such that the restriction of Theorem 2 is accomplished. No particular emphasis was given on the tuning process, since the resulting performance with these simple parameters is very acceptable. The attitude is maintained as a quaternion, which is normalized at each time step to compensate for numerical errors. After that, the quaternion is converted to Euler Angles, since they are more intuitive, and it allows for comparing with the angles obtained from the Twin Rotor System. For the entire experimentation time span, the vector *v⃗_f_* = *v⃗_o_* + *v⃗_r_* was added to the vector of the rate gyro measurements, in order to test the ability to estimate and compensate for the rate gyro bias. In this case, one has *v⃗_o_* = 0.1(1 – 1 1)*^T^* and *v⃗_r_* = 0.0001*k*(1 1 1)*^T^*, where *k* represents the iteration index.

**Figure 7.**
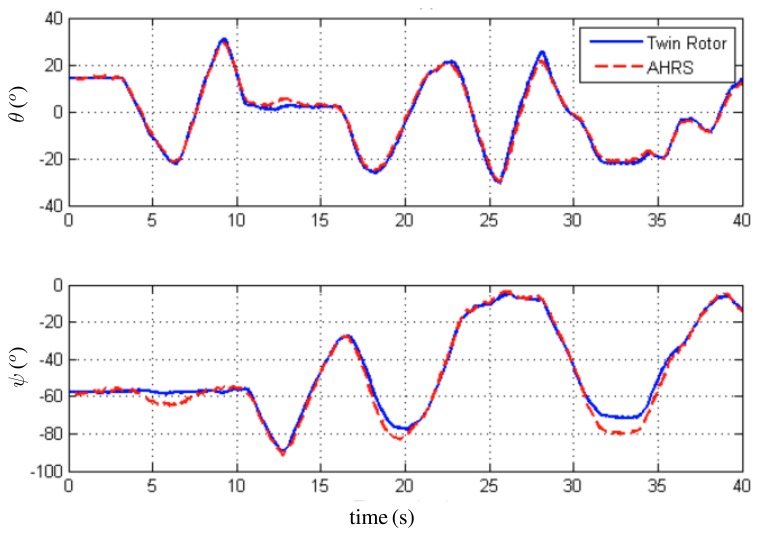
Estimation of *θ* (pitch) and *ψ* (yaw) angles.

**Figure 8.**
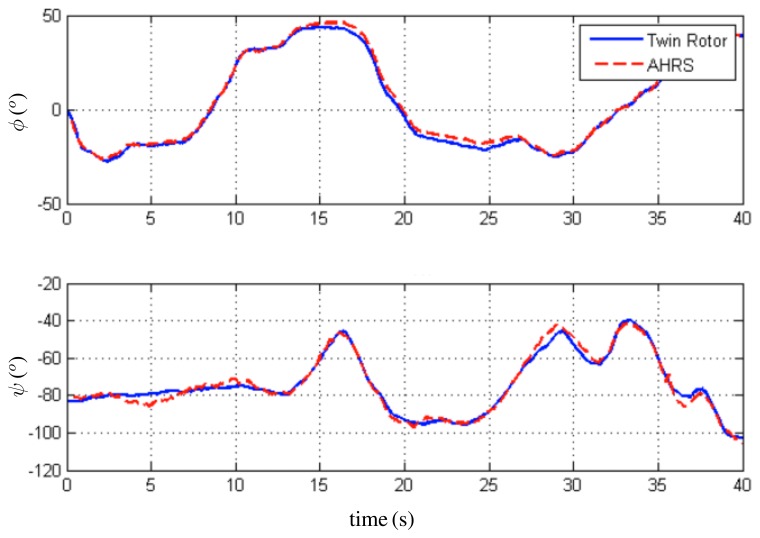
Estimation of *Φ* (roll) and *Ψ* (yaw) angles.

**Figure 9.**
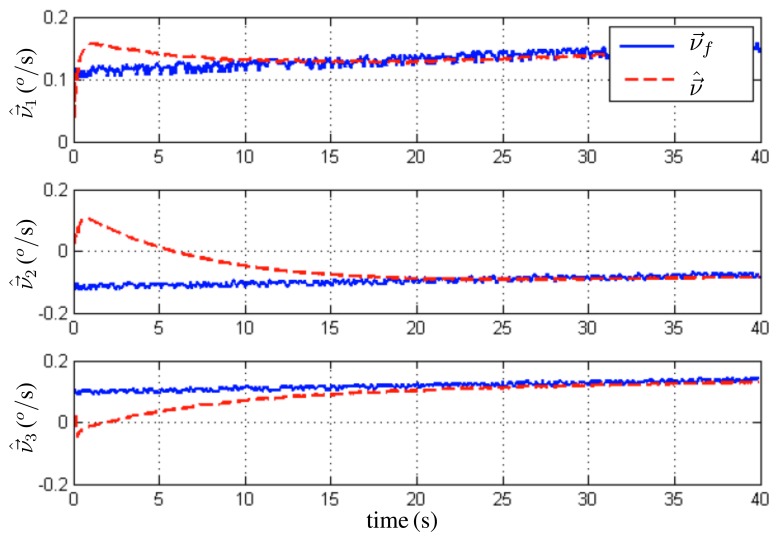
Evolution of the rate gyro bias estimate.

For the first trial, the initial condition for the quaternion estimate is *q⃗*(*t*_0_) = (0.85 0.06 0.11 – 0.49)*^T^*, *i.e.*, (*ϕ̂* = 0°, *θ̂* = 15°, *ψ̂* = −60°). For the second trial, the initial condition for the quaternion estimate is *q̂*(*t*_0_) = (0.76 0 0 - 0.64)*^T^*, *i.e.*, (*ϕ̂* = 0°, *θ̂* = 0°, *ψ̂* = −80°). In both cases, the rate gyro bias initial estimate was set to zero. The observer angle estimates are compared with the ones obtained from the encoders of the Twin Rotor System. In Figure 7, the evolution of *θ* (pitch) and *Ψ* (yaw) angles is depicted. Figure 8 shows the evolution of the second trial, *i.e.*, *Φ* (roll) and *Ψ* (yaw) angles. Finally, the convergence of the rate gyros bias estimates is shown in Figure 9. These results indicate that the observer faces up to the slowly time-varying nature of the rate gyro biases.

**Figure 10.**
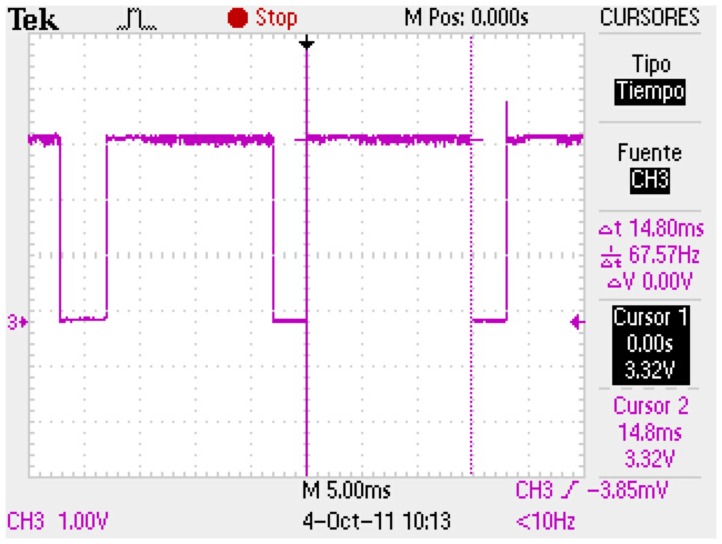
Algorithm CPU usage.

It is worth mentioning that the algorithm itself uses only 81% of the 55 Hz cycle time. This is depicted in Figure 10. Finally, we invite the readers to watch the following video: https://www.dropbox.eom/s/r3pq57rwlf0pxuv/AttitudeObserver.mov.

## 8. Conclusions

This paper presented an original quaternion-based strategy for the attitude estimation of a rigid body equipped with angular velocity sensors and reference vector sensors. To accomplish the objective, a robust nonlinear observer for attitude estimation was developed. This observer ensures the asymptotic estimation convergence and local exponential estimation convergence. Furthermore, by means of an ISS analysis, it was shown that the observer is robust towards noise and the disturbance of the rate gyro sensors. Thus, an embedded AHRS was designed and developed. Experimental results were presented, where the AHRS outputs are compared with a two degrees-of-freedom high accuracy motion system. This illustrates the achievable performance of the proposed estimation schema.
